# Implementation considerations in case-area targeted interventions to prevent cholera transmission in Northeast Nigeria: A qualitative analysis

**DOI:** 10.1371/journal.pntd.0011298

**Published:** 2023-04-28

**Authors:** Gurpreet Kaur, Lindsay Salem-Bango, Ana Leticia Melquiades dos Santos Nery, Emmanuel Chimda Solomon, Emmanuel Ihemezue, Christine Kelly, Chiara Altare, Andrew S. Azman, Paul B. Spiegel, Daniele Lantagne

**Affiliations:** 1 Center for Humanitarian Health, Johns Hopkins Bloomberg School of Public Health, Baltimore, Maryland, United States of America; 2 Action Contre Le Faim, Maiduguri, Borno, Nigeria; 3 Solidarités International, Maiduguri, Borno, Nigeria; 4 Tufts University School of Engineering, Medford, Maryland, United States of America; 5 Department of Epidemiology, Johns Hopkins Bloomberg School of Public Health, Baltimore, Maryland, United States of America; The University of Kansas, UNITED STATES

## Abstract

Cholera outbreaks primarily occur in areas lacking adequate water, sanitation, and hygiene (WASH), and infection can cause severe dehydration and death. As individuals living near cholera cases are more likely to contract cholera, case-area targeted interventions (CATI), where a response team visits case and neighbor households and conducts WASH and/or epidemiological interventions, are increasingly implemented to interrupt cholera transmission. As part of a multi-pronged evaluation on whether CATIs reduce cholera transmission, we compared two organizations’ standard operating procedures (SOPs) with information from key informant interviews with 26 staff at national/headquarters and field levels who implemented CATIs in Nigeria in 2021. While organizations generally adhered to SOPs during implementation, deviations related to accessing case household and neighbor household selection were made due to incomplete line lists, high population density, and insufficient staffing and materials. We recommend reducing the CATI radius, providing more explicit context-specific guidance in SOPs, adopting more measures to ensure sufficient staffing and supplies, improving surveillance and data management, and strengthening risk communication and community engagement. The qualitative results herein will inform future quantitative analysis to provide recommendations for overall CATI implementation in future cholera responses in fragile contexts.

## Author summary

Infecting millions of people every year, cholera is a deadly water-borne disease that particularly plagues resource-limited settings. Communities facing humanitarian crises experience even greater challenges in preventing and responding to cholera outbreaks. A recent and increasingly common strategy in cholera outbreaks is case-area targeted interventions (CATIs). Rather than target an entire community in a blanketed approach, CATIs target the case household and surrounding neighbor households within a specified radius with WASH supplies and other activities. However, there is limited evidence about the challenges and effectiveness of this strategy, especially in fragile contexts. This study interviewed rapid response team members in Northeast Nigeria (Borno, Adamawa, and Yobe states) implementing CATI during a 2021 cholera outbreak. It identifies challenges that led teams to deviate from standard operating procedures and provides recommendations for future CATI implementation. This new knowledge may contribute to the development of evidence-based CATI protocols in future cholera outbreaks in humanitarian settings.

## Introduction

Infection with toxigenic *Vibrio cholerae* O1/O139 bacteria can cause profuse watery stool, vomiting, and severe dehydration, and can, without prompt medical treatment, result in death [1]. Endemic cholera is more likely to occur in areas lacking adequate water and sanitation services [2]. In 2020, 27 countries reported 323,320 cases of, and 857 deaths from, cholera [3]. The total global cholera burden is estimated to 2.9 million cases and 95,000 deaths per year [4]. The Global Task Force on Cholera Control (GTFCC) aims to reduce cholera deaths by 90% by 2030 [5].

While cholera transmission is known to occur via contaminated food and water, there is a growing body of evidence suggesting person-to-person transmission within households and to neighbors is an important transmission route. Recent research has documented individuals living within 50 meters of a cholera case are 23–56 times more likely to contract cholera than those further away [6], and mean infection risks of 3.7–8.2% were associated with fecal shedding of *V*. *cholerae* among household contacts of cases, compared to infection risks of 2.0–3.4% from community water sources [7].

To reduce intra-household and neighbor transmission, case-area targeted interventions (CATIs) are a recent and increasingly commonly implemented approach [8,9]. CATIs are performed by a team traveling to the household of an admitted cholera patient that conducts interventions intended to interrupt transmission at case and neighbor households to extinguish or reduce the spread of the overall outbreak. While specific CATI packages vary across contexts, they can include *water*, *sanitation*, *and hygiene* (*WASH) interventions*, such as the distribution of soap, water storage containers, and water treatment supplies; treating community water supplies; household spraying; and hygiene promotion; and *epidemiological interventions*, such as active case finding, supportive care, vaccination, chemoprophylaxis, and health education [10].

There is some evidence that individual CATIs are effective mechanisms to reduce cholera transmission and a spatiotemporal ring of 50–100 meters is appropriate, depending upon the context [10]. There is also emerging evidence that CATIs may reduce case-cluster sizes (in Haiti) and infection among household contacts (in Bangladesh), and there are suggested reductions in cholera transmission in post-intervention Cameroon and Democratic Republic of Congo [10]. However, we currently know of no published randomized controlled CATI evaluations. In particular, there is a research gap in understanding the effectiveness of different packages of CATIs, as actually implemented, in a prospective trial design.

To build on this emerging evidence base, the Center for Humanitarian Health at the Johns Hopkins Bloomberg School of Public Health, with funding from USAID/BHA, conducted prospective impact evaluations of CATI implementations in humanitarian contexts, with a planned impact measure of reduced cholera transmission. As part of the overall evaluation design, the package of CATIs as delivered was investigated, including reviews of standard operating procedures (SOPs) and qualitative key informant interviews (KIIs) with CATI delivery staff. These data facilitate the understanding of how CATIs are actually completed in fragile contexts to provide recommendations for implementation, and they provide important context to forthcoming quantitative analyses.

## Methods

### Ethics statement

These KIIs were approved by the Institutional Review Board (IRB) at the Bloomberg School of Public Health (#14535). Nigeria state-specific IRB approvals from Borno and Yobe were obtained for SI and ACF, and Adamawa IRB approval was obtained for SI. Tufts University IRB issued a non-research determination for the analysis completed by Tufts on de-identified data only. Formal verbal consent was obtained from participants.

The 2021 Nigeria cholera outbreak impacted the crisis-affected Northeast States of Borno, Adamawa, and Yobe, which reported suspected cholera case numbers of 6,353, 1,945, and 4,016, respectively [11]. Action Contre la Faim (ACF) and Solidarités International (SI) implemented the CATI approach during cholera response activities, with ACF implementing in Borno and Yobe, and SI implementing in Borno, Adamawa, and Yobe.

The Bloomberg School of Public Health partnered with ACF and SI separately to conduct prospective impact evaluations of their CATI implementations. The evaluation included both qualitative and quantitative components. For this qualitative analysis, we reviewed and compared ACF and SI’s SOPs with information gained from KIIs with CATI teams and management/headquarters staff. The quantitative analysis is ongoing at the time of publication.

KIIs were held with ACF and SI staff involved in the response from December 2021 to March 2022. The KIIs covered two groups: 1) staff responsible for implementing CATIs (‘Field KIIs’); and 2) managers, including headquarters-based technical advisors responsible for developing the CATI SOPs, staff trainings, and cholera response coordination (‘Management/Headquarters KIIs’).

#### CATI team KIIs

A 25-question semi-structured KII guide was developed to understand field realities of CATI implementation in cholera responses. The questions explored team composition, case identification, supplies, intervention delivery, radius measurement, neighbor household selection, challenges, and future recommendations. KII participants were identified and invited to attend by their respective ACF and SI field-based line managers. The KIIs were held in groups of 2–7 individuals, based on logistics. During KIIs, de-identified mapped CATI Rings (from the spatial analysis conducted for the quantitative portion of this research) were used as visual guides.

#### Manager KIIs

A 28-question semi-structured KII guide was developed to explore macro-level perceptions of the cholera response, coordination, CATI delivery, and implementation. ACF and SI participants were identified by their respective NGO leads as key actors in the cholera response and invited to participate. Manager KIIs were held individually or in pairs.

Interviews were conducted over Zoom (San Jose, CA, USA) or Microsoft Teams (Redmond, WA, USA). The interviews were held in English and recorded with participant verbal consent. Auto-transcriptions were cleaned and uploaded into Dedoose (Manhattan Beach, CA, USA) for qualitative analysis.

Qualitative content analysis was used because the KII guide included open-ended questions. All transcripts were coded in Dedoose. Themes were coded independently for management versus field KIIs and reported within these two classifications. Themes evolved naturally from the transcripts and were mutually reviewed and agreed upon by the research team to create a master list of codes. These final codes were applied to each interview by one of two research team members then cross-checked for consistency. Differences in coding were resolved by consensus. A sub-sample of the coded transcripts were subsequently reviewed by an external third research team member for additional quality control.

In the results section, first the SOPs are summarized, and then KII results are presented by SOP section and compared with SOPs to note alignments and deviations from planned SOPs. Percentages in the results were calculated using the number of unique respondents mentioning a specific challenge as the numerator and total number of respondents mentioning any challenge in the denominator. This was done at the aggregate level (total respondents) as well as disaggregated by group type (field- versus management-level KIIs). For the numerator, the same challenge reported several times by the same individual was only counted once. In group interviews, respondents who spontaneously mentioned a challenge were counted, as were respondents who spontaneously agreed. Polls were not taken to get a systematic count of agreement from remaining participants in the interview. For the denominator, only respondents that reported a challenge to the posed question were included in the total count; if a respondent did not report any challenge to the specific question, they were not included in the calculation.

## Results

### SOP review

ACF and SI SOPs detailed the makeup of CATI teams implementing each CATI, how to acquire a list of case households to receive interventions, actions to complete at the cholera case households, selection of neighbor households, actions to complete at neighbor households, and post-intervention monitoring. Please note these SOPs are available from authors upon request.

#### CATI teams

Both ACF and SI SOPs describe the CATI team as consisting of 2–4 members who have a WASH and/or health background, possess good communication skills, and were from the same Local Government Area (LGA) as the cases. Either individually, or in combination, the CATI team fills these roles: 1) a Team Leader that oversees each CATI implementation; 2) hygiene promoters that explain cholera symptoms, transmission routes, and prevention practices; and 3) WASH Technicians that disinfect households and treat water sources.

#### Acquiring case household list

Both ACF and SI SOPs stated team leaders should physically go to cholera treatment centers (CTCs) and health facilities to obtain the patient names and addresses to receive CATI interventions. The SI SOP also stated that teams can receive patient information electronically via WhatsApp, text, phone, or email. ACF SOPs state interventions should be conducted within 1–2 days of cholera patient admission, while SI SOPs describe an intervention window of up to 3 days after admission.

#### Case household interventions

Both SOPs stated that the CATI implementation should begin with team introductions at the household, followed by disinfection of the household with chlorine solutions, then distribution of cholera kits and promotion of hygiene behaviors. Specifically, team supervisors should introduce the team to the case household members, explain the activities to be conducted, and obtain consent before continuing. The ACF SOP noted additional steps of introducing the team to community leaders (and inquire about any possible concerns about stigma about cholera) to seek permission to implement before proceeding to the case household.

Both SOPs stated that two concentrations of chlorine solution should be used during household disinfection, including 0.2% (for the patient bed and other soiled items) and 2.0% (for latrine and bathing areas). Additionally, surfaces should be sprayed until wet, with a thin layer of observable chlorine. The SI SOP further included spraying the inside, then outside of shelter walls with 0.2% chlorine from the top to bottom of each wall, and that patient clothes and bedsheets should be soaked in a bucket with 0.2% chlorine for 15 minutes and dried in the sun. ACF’s procedure stated these items should be soaked in local laundry detergent and dried in the sun.

After spraying, both SOPs stated that hygiene kits should be given to the household and hygiene promoters should demonstrate how each item should be used. The procedure from SI stated that kits should include 200 Aquatabs, 18 bars of bathing soap, 18 packets of laundry powder, a kettle with a lid, a 1 L cup, a 20 L jerrycan, a 20 L bucket with a lid, and a cholera prevention flyer. While this amount could be adjusted, case households should receive enough materials for 2 months of use. The procedure from ACF stated that case households should receive cholera kits including 10 sachets of oral rehydration salts per person, 2 bars of soap per person, 30 days of water treatment (e.g. Aquatabs), and an improved water container. They should also receive a hygiene kit including a bottle of commercial bleach, a sachet of laundry detergent, a bucket, a cleaning cloth, and a scrubbing brush.

The SOPs then explained that teams should give hygiene promotion messages to household members, which should include cholera prevention practices, potential transmission routes, general awareness, and hygiene practices. Lastly, if feasible and relevant, CATI teams from both organizations should investigate contextual reasons for cholera transmission and treat drinking water from case household and the community water sources.

#### Selection of neighbor households

Per ACF and SI SOPs, neighbor households falling within a defined radius around the case household (the ‘CATI ring’) also should receive targeted interventions. Both organizations’ SOPs included a recommended range, with the exact radius to be selected by each country. ACF Nigeria determined a radius of 150 meters from a recommended 50-150-meter range. SI Nigeria also selected 150-meters from a recommended 30-150-meter range, depending on density and dispersion of neighbor households.

#### Neighbor household interventions

Both SOPs included hygiene promotion activities for neighboring households within the CATI ring, where teams should explain the symptoms of cholera, transmission routes, and protective behaviors. The SI SOP also stated all households within the CATI ring should be disinfected following the same procedure used in the case household, and neighbor households should be given 8 bars of soap and 100 tablets of Aquatabs, sufficient to last one month. The ACF SOP outlined the provision of aforementioned cholera kits to neighbor households but did not mention neighbor household disinfection.

#### Post-intervention monitoring

Both SOPs described post-intervention monitoring, including utilizing case and neighbor household coordinates to track outbreak response and monitoring key performance indicators for CATI such as delay of interventions and number of case households disinfected.

### Key informant interviews

A total of seven group KIIs were conducted among ACF and SI CATI teams. The KIIs were conducted separately by NGO and location: five CATI team leaders and four supporting team members (hygiene promoter/disinfector) were interviewed from ACF; eight CATI team Leaders and one CATI team member were interviewed from SI. Six management-level KIIs were conducted across ACF and SI. Four individuals from each organization were interviewed across the spectrum of WASH technical advisor, WASH country mission lead, cholera technical lead, and/or WASH emergency program manager. As noted in Methods, these interviews were conducted individually or in pairs.

We present KII results and planned SOP compared to actual implementation.

#### CATI teams

In practice, CATI teams included a team leader/supervisor who collected patient information and instructed the team; hygiene promoters who informed CATI recipients about the symptoms, transmission, and prevention of cholera; and WASH Technicians who conducted disinfection. SI hired team members directly, while ACF CATI team members were primarily volunteers from the government rapid response team paid per diem by the NGO. An enumerator responsible for the study’s data collection was either an additional member of the team or this role was assigned to the CATI team leader/supervisor. Overall, team composition and roles (as reported by ACF and SI key informants) were consistent with SOPs.

#### Acquiring case household lists

CATI teams obtained case information generally following the SOPs. For ACF and SI CATI teams, case notification occurred in the morning at partner CTCs (run by the Ministry of Health or other NGOs) when the CATI team leader/supervisor identified case households from case line lists. Additionally, an ACF team in Borno and an SI team in Adamawa posted staff at the CTC to update CATI teams of new case admissions during the day, relaying patient information by direct communication, phone call, or WhatsApp to CATI teams. Contact information obtained from the patient list included the case name, phone number, address, and/or household location description.

Decisions on which case households to visit were determined by the CATI team leader/supervisor. Teams sometimes tried to call the case household before visiting. A single CATI team visited 1–5 cases per day (average 2–3) depending on location and epidemic case count. CATI teams reported days when they were unable to respond to all new case admissions, particularly during the peak of the outbreak. On these exceeding capacity days, households were selected for CATI implementation based on criteria such as proximity of case households to other cases, population density, and team knowledge of the household location.

*“Whenever there are huge number of cases at the CTC*, *the team lead from the team who went to receive the cases picks a certain number of cases since they won’t be able to attend to all*.*…based on the communities with high number populations*.*”*

– Field Staff/Volunteer

#### Case household Interventions

ACF and SI informants reported providing similar case household interventions. Upon arriving at a case household, the hygiene promoter introduced the team and its purpose, and requested household permission to conduct CATI. Once introductions were complete and consent acquired, the hygiene promoter provided WASH messaging to household members while the sprayer disinfected the household.

Two concentrations of chlorine solution were used during disinfection: 2% and 0.2%, per SOPs. CATI team members from SI disinfected latrines and bathing areas with 2% chlorine solution and the cases’ walls and bedding with 0.2% chlorine. A WASH Technician from ACF reported spraying 2% chlorine solution on materials in case households and 0.2% chlorine solution on materials in neighboring households.

Hygiene promotion messaging included: causes of cholera; factors that influence its spread; and prevention measures like handwashing, covering food, environmental cleanliness, and Aquatab use. Each case household also received a cholera kit. Both ACF and SI provided soap and Aquatabs. ACF also provided a rechargeable solar torchlight, oral rehydration salts, drinking cups, one kettle, two 20 L jerrycans, and one 25L bucket. SI also provided a bucket, one jerrycan, a kettle, and drinking cups.

In total, hygiene promotion took 10–15 minutes and spraying 5–10 minutes. Depending on the size of the house, CATI teams spent a total of 15–30 minutes at each case household. The CATI team moved together, with sprayers waiting until hygiene promoters were finished before moving to the next house.

*“There was the classic set up with the teams doing the spraying in the high risk areas—in the toilets*, *in the kitchen*, *in the bedding and I think sometimes also in the house*. *There was a hygiene kit*. *There was a hygiene promotion*. *And they chlorinated the household water supply*. *This this was done always*.*”*

– Field Staff/Volunteer

*“[F]irst of all*, *we get there—we introduce ourselves*, *the organization we are from*, *and the reason of our coming to their house*. *Then we give them the hygiene promotion*, *lectures*, *then we give them the kits*, *which comprises of the jerrycan the bucket*, *the Aquatabs and how to use them*. *The soaps and the detergent*… *we distribute it to them—then*, *afterwards*, *the sprayers*….*”*

– Field Staff/Volunteer

According to the KIIs, ACF and SI were generally aligned with their SOPs when introducing team members to the households and distributing hygiene messaging, and in total time spent in case households. The actual number of Items distributed in cholera kits mostly aligned with the SOP but varied at times (see stock outs section below). Additionally, there were slight variations between how chlorine disinfection was intended to be completed and how it was actually completed, with staff reporting spraying case households with 2% chlorine and neighbor households with 0.2% (instead of using different chlorine solutions for different items).

**Fig 1:**
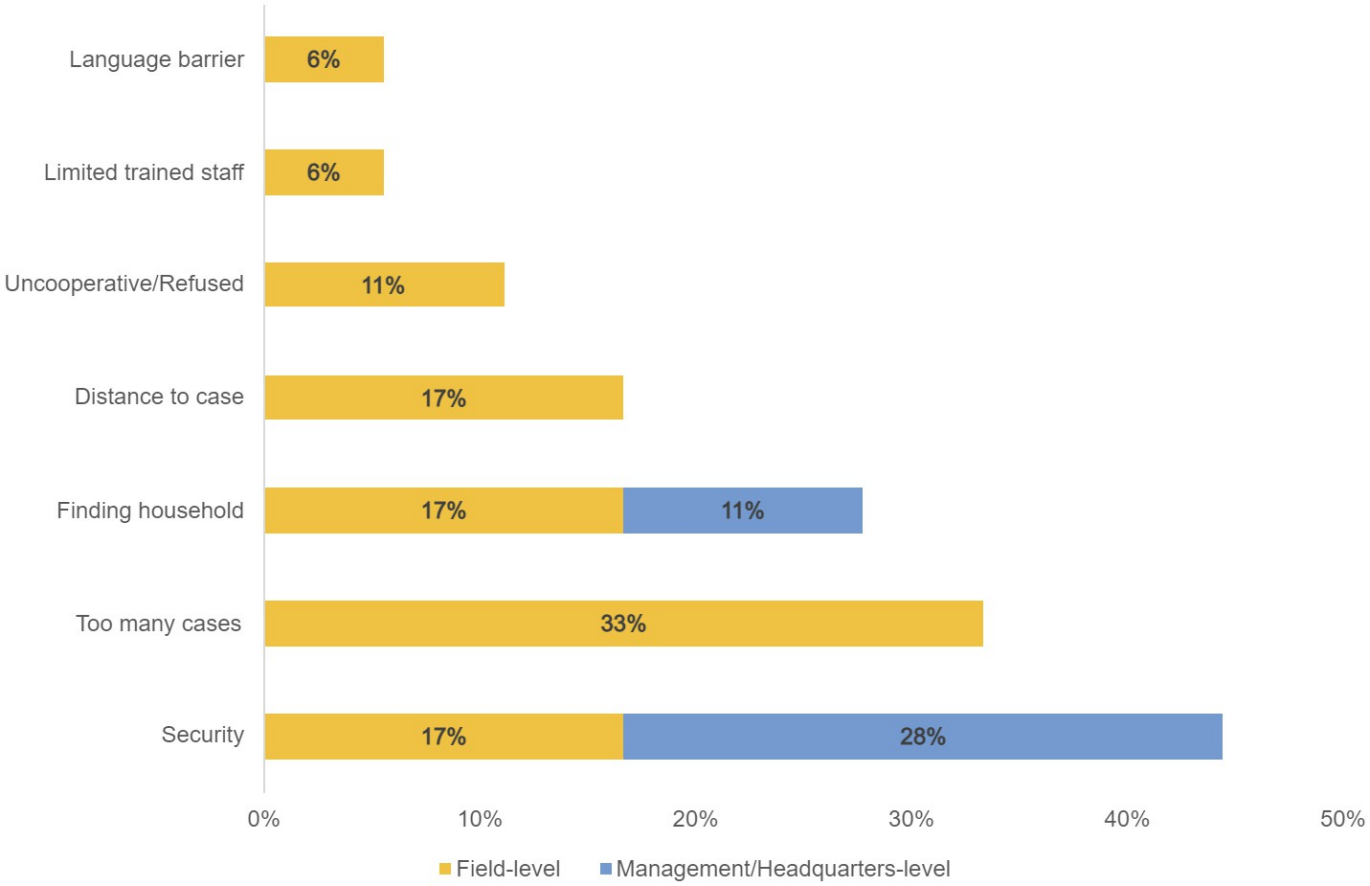
Frequency of challenges related to the implementation of CATI at the case household. This figure shows the frequency of spontaneously cited challenges related to the implementation of CATI at case households, categorized by field- and management-level respondents (n = 18). The numerator is the number of unique mentions of a specific case-related challenge by field- or management-level respondents. The denominator is the total number of field- or management-level respondents who cited case-related challenges. For example, 6% of all respondents reporting challenges at case households mentioned language barriers (all among field-level respondents) and 45% mentioned security (from both field- and management-level respondents).

One commonly cited challenge was finding the case household (28% of challenge mentions across field and management KIIs), as case household addresses sometimes lacked sufficient detail, especially in rural or semi-rural areas (Fig 1). If the CATI team could not locate the house, the team attempted to call the phone number from the CTC line list (if available) to obtain directions. If cases did not have a working phone, or did not answer their phone, ACF staff mentioned attempting to contact family members and/or asking community members for directions to the case household. After these attempts, if the case household could not be identified, the teams moved onto the next case household on the list.

*“OK*, *so one of them [challenge] was then they had serious issues getting the addresses*, *phone numbers of this case households*. *It’s like in the health facility*, *they were really not documenting properly*. *And so you—they were having challenges locating the case household*. *And sometimes they might be discharged*. *And so to locate that house*…*pose[s] a serious issue*. *“*

– Management/Headquarters Staff

Another commonly cited challenge was security issues (particularly in Borno) that precluded reaching some case households (45% of mentions across field and management KIIs) (Fig 1). CATI teams were also denied access to the rehabilitation center for former insurgents in Maiduguri, even though cholera cases from the rehabilitation center were treated at the CTC.

*“Like*, *when we get to a particular village*, *I will tell them that we should be watchful of our time not exceeding after 3pm*. *Because as at that time*, *that’s when most of these things happen*. *Okay*, *so we normally leave the place before that time*.*”*

– Field Staff/Volunteer

Another challenge reported by ACF CATI teams was large case load (33% of challenge mentions). Teams in both ACF response areas reported that, particularly during the outbreak peak, they sometimes did not have sufficient staffing or time to respond to all cases, as described above.

SI field staff also reported that cases occasionally refused CATI team visits (11% of mentions) (Fig 1). Some cases rejected CATI after introductions, and others reportedly hung up or refused to answer team members calling for directions. However, this does not appear to have happened often, although quantitative data on the number of households rejecting are not available from these qualitative data from individual informants. Other lesser reported challenges included distance to case (17%), limited trained staff (6%), and language barrier when responding to case households (6%).

#### Neighbor household selection

Both ACF and SI CATI teams visited neighbor households within a theoretical 150-meter radius from the case household for CATI implementation. While in theory all consenting neighbor households within that radius should have received CATIs, the implementation varied for both organizations.

Both ACF and SI used 300 footsteps as a proxy measure for the 150-meter radius. Rather than work in a true radius, CATI team leaders/supervisors often identified the last neighbor household in each cardinal direction to the team; neighbor households falling within that distance were approached to receive CATI interventions. Roads falling within the CATI coverage area were addressed based on context. In some instances, if the road was congested and/or lined by non-residential structures such as shops, the CATI team did not cover neighbor households on the other side of the road. In other instances, CATI teams crossed the road and accounted for road length in the neighbor household radius.

*“So now*, *when I cross the road*. *Now*, *for instance*, *if the road is 10 meters from the case house and my intention was to go 40 meters*. *I will reduce it*. *Because the distance from the case household and to the road yes*, *kind of break*…*that distance that I was supposed to have taken*.*”*

– Field Staff/Volunteer

If teams noticed CATI rings overlapped, they adjusted their neighbor response based on response time between case households. If two cases occurred close together <72 hours apart, teams visiting the second case sometimes only visited additional neighbor households upon neighbor request. Other times, they visited neighbor households immediately surrounding the case household, but not further. If cases occurred >72 hours apart, teams treated the case as a new CATI response. Teams were unconcerned about unintentionally targeting the same household twice, as teams typically were unable to reach every household in the first CATI ring.

Team members reported appreciating the 150-meter radius, citing research it might contribute to reduced transmission. However, the practical application of the 150-meter radius was reported as challenging for a variety of reasons, including changing radius guidelines, physical barriers, trade-offs between reaching more neighbors or additional case households because of population density or supply shortages, and insufficient tools for measuring distance.

While SI and ACF SOPs had a theoretical 150-meter radius from case household, CATI team training varied. For example, SI staff were initially trained to use a 30-meter radius. This was changed to 150 meters after training, with the updated radius communicated to teams, but no new training occurred. Managers suggested this may have caused confusion, especially in the beginning of the response.

Limited human and physical resources also hindered the radius’ implementation within both organizations. Interviewees at both the field- and headquarters-level at SI and ACF stated there was inadequate staffing to respond to this proposed coverage area, particularly in areas with high population density. A high household count also quickly depleted CATI supplies; stock outs are discussed in more detail in *Neighbor household implementation*.

*“We have some locations where you have*, *it’s a dense population… [I]n the same 50 by 50 feet ideally for one household*, *we find out that there are close to 10 households in the same 50 by 50…*. *So*, *instead of you just seeing one household in that land area*, *you have to*, *probably have to deal with close to like*, *more than you have expected*, *10*, *sometimes 15 sometimes*. *So*, *but but then—it didn’t in any way change that fact that yes*, *we still needed to see how we can physically be able to reach to have this radius of 150 meters*.*”*

– Management/Headquarters Staff

SI staff reported needing to make trade-offs between reaching more neighbor households (within a single CATI), or responding to more case households (i.e. doing more CATIs). With a 30-meter radius, a single team reportedly visited seven case households per day. When the radius increased to 150 meters in dense populations, this dropped to 2–3 case households per day per team.

*“[W]hen we began to push the team [to reach 150 meters]*, *we noticed a decline in the number of cases they’re able to meet*. *And then we were able to train and deploy more teams to be able to respond*.*”*

– Management/Headquarters Staff

To address highly dense areas, SI began using the 300-step radius and stopped responding when they ran out of supplies or time, while ACF transitioned over time to using a household count proxy based on estimating household plot size. After reaching the determined number of households, they considered the ring complete. Teams within both organizations typically spent 1.5 to 4 hours responding to neighbor households in each CATI ring.

*“For this case*. *It’s a very dense population*. *So definitely they take like 15 households*, *5 households at the front*, *five at the left*, *five at the right*, *5 at the back*, *making 20 and any other households around specifically*.*”*

– Field Staff/Volunteer

ACF and SI CATI teams reported it was difficult to accurately assess their coverage as they had no measurement or mapping instruments beyond counting steps. In fact, interviewees thought they were appropriately covering the full radius, until KII administers showed them maps of actual implemented CATI rings. To assist in ensuring adequate coverage, CATI teams recommended they have access to mapping technology to assist with determining radius coverage.

*“Because even me presently I’m really a bit surprised to see how big the circle is*. *150 meters are very big*.*”*

– Field Staff/Volunteer

*“For us*, *it was going fine with the teams we had*. *Not until we were told that okay*, *with the data that has been collected*, *they are not able to cover the 150 meters radius*.*”*

– Management/Headquarters Staff

Informants had mixed views on changing radius length. The most common viewpoint was that the 150-meter radius was too large for the resources available and would only be possible with more or larger CATI teams. Other informants suggested determining the radius based on population density, with the distance or targeted number of households varying by context.

Some informants, however, recommended keeping the radius length at 150 meters, with one informant expressing concern about reaching sufficient neighbor households when accounting for refusals. Another suggested the challenges were because this was the first time implementing a radius this large. Only one informant suggested increasing the radius length.

*“If we’re talking of that… 150 meters radius*, *it’s a lot of households*. *It’s a lot of spaces to cover*. *So we need to have more human resources and materials*.*”*

– Management/Headquarters Staff

*“Okay*, *let’s but obviously*, *if I may*, *if I have to*, *from what I’ve seen from when I follow the study for the different maps that you show us*… *I will say that the 150 meter is probably it’s probably too much*, *maybe we should make maybe*, *maybe the 30 meters was too little*? *And something around maybe between 50 to 100*?*”*

– Management/Headquarters Staff

*“I think that it’s obviously depending if we are on a dense urban setting or in a rural areas [sic] or in between*, *you won’t you won’t have the same perspective*. *So definitely*, *I mean*, *I think you have different [settings] you have to adapt to*. *And that could be interesting maybe to to [sic] make some recommendation based on the on the density*.*”*

– Management/Headquarters Staff

#### Neighbor household implementation

Across both ACF and SI, the main interventions at neighbor households in a CATI ring consisted of distribution of soap and Aquatabs, disinfection, and hygiene promotion. The quantities of soap and Aquatabs provided varied by state and organization. ACF CATI teams in Yobe provided 2 pieces of soap and 5 Aquatabs to neighbor households, and 5 pieces of soap and 200 Aquatabs in Borno. SI CATI teams in Borno provided 8 pieces of soap and 10 Aquatabs. Sometimes, information, education, and communication (IEC) posters were provided to neighbor households.

In agreement with their SOP, SI CATI team members reported introducing themselves to neighbors, asking for their consent to conduct an intervention, and discussing hygiene promotion such as methods of cholera transmission and prevention, handwashing, and water chain management, disinfecting their household following the same procedure as the case household, and distributing soap and Aquatabs. These steps were also reported by ACF team members but detailed methods of conducting neighboring households beyond hygiene promotion were not listed in their SOP. SI’s disinfection of neighbor households aligned with their SOP. ACF teams disinfected neighboring households but the SOP did not instruct them to. Hygiene promotion was conducted following the SOP although the number of Aquatabs and soap bars distributed were different from the SOP.

Informants reported it took CATI teams 5–15 minutes (depending on household size) to complete activities at one neighbor household.

*“It takes 15 minutes or 10–15 minutes at the case house*. *It usually takes less than that to conduct it in the neighboring house*.*”*

– Field Staff/Volunteer

**Fig 2:**
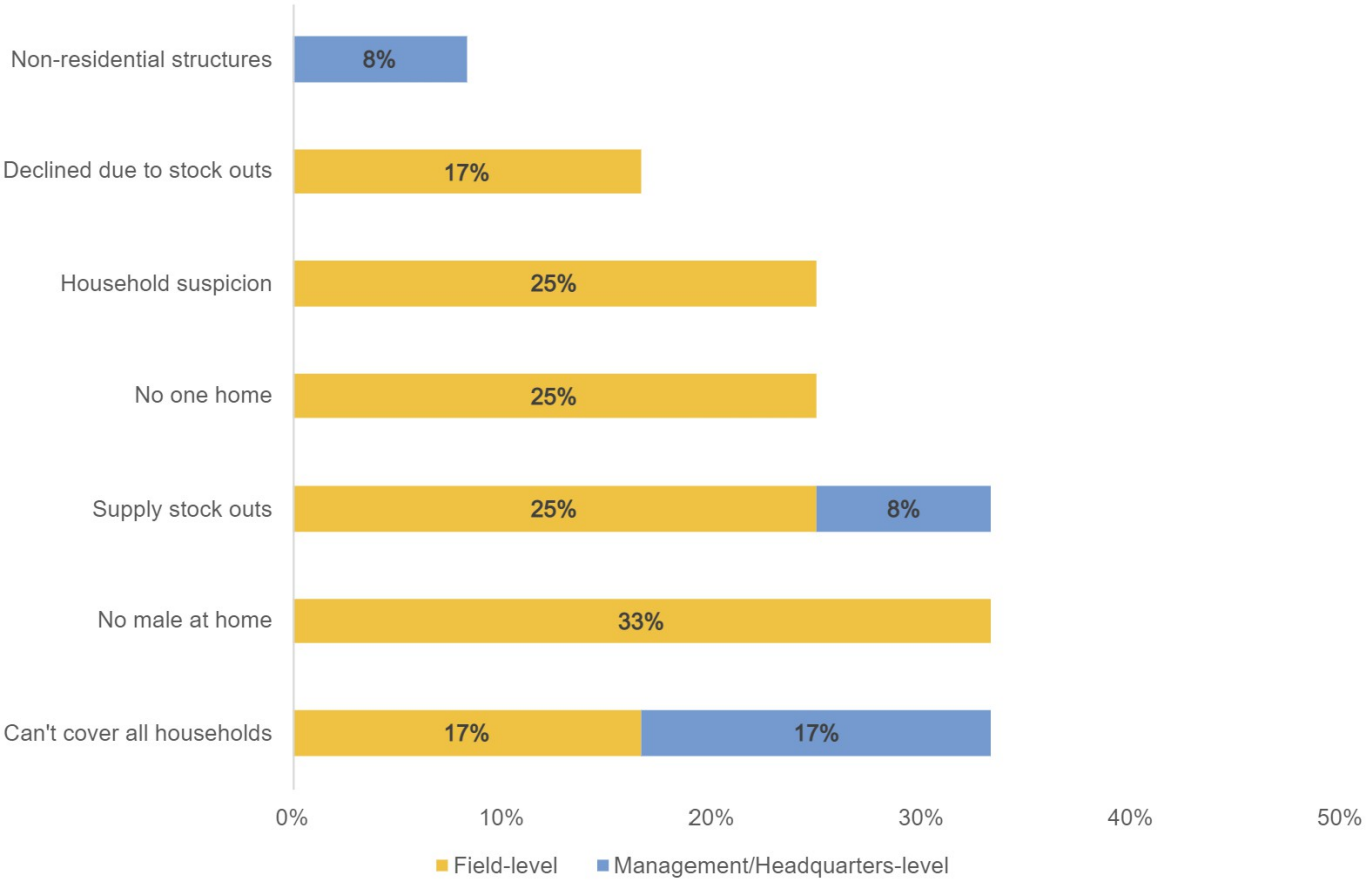
Challenges related to the implementation of CATI at the neighbor households. This figure shows the frequency of spontaneously cited challenges related to the implementation of CATI at neighbor households, categorized by field- and management-level respondents (n = 12). The numerator is the number of unique mentions of a specific neighbor-related challenge by field- or management-level respondents. The denominator is the total number of field- or management-level respondents who cited neighbor-related challenges. For example, 34% of all respondents reporting challenges at neighbor households mentioned being unable to cover all households; half (17% of all respondents) were field-level.

The most common challenges reported by SI and ACF CATI teams in responding to neighbor households (excluding radius-related challenges as described above) included: supply stock outs (33%); refusal because the male head of household was not home (33%); and inability to cover all neighbor households due to team capacity (33%) (Fig 2). Other common challenges reported by field staff included household suspicion of the intervention (25%) and no one home during the visit (25%). Some CATI field teams also reported neighbor households declined interventions specifically because of supply stock outs (17%) and management-level staff noted that non-residential structures falling within the 150-meter radius challenged CATI coverage (8%).

*“Some people used to say that the males are not allowed to enter inside a married woman environment*, *house*. *When we went there some the husbands are not around … so they [the wives] will not give us the permission to enter the house*. *That is the major reason*.*”*

– Field Staff/Volunteer

The Management KIIs stated similar challenges as CATI teams (Field KIIs) in regards to covering all neighbor households within the 150-meter radius. Additionally, Management KIIs noted stockouts, but could not distinguish whether it was a field stock out (at the CATI team level) or a central warehouse stock out (at the project level). ACF and SI KIIs indicate supply stock outs of Aquatabs, soap, and/or IEC material were an issue more at neighbor households than at case households. Stock outs at the community level seemed mostly attributed to transport capacity, affecting both case and neighbor households; these could be corrected by sending the transport back to the warehouse to replenish supplies while the CATI team continued its interventions. Stock outs at the project level (the warehouse) affected the soap and Aquatab supplies provided to neighbor households to a greater extent than the cholera kits supplied to case households.

*“If we run out of supplies*, *normally we get to the household*. *We make sure that there is chlorine*, *because chlorine is never out of her supply*. *Yeah*, *we just get there to disinfect*, *give them the hygiene promotion and then just move”*

– Field Staff/Volunteer

*“Actually*, *it was a bit erratic*. *Let’s say*, *we experienced this shortage for probably one week*, *two weeks*, *and then we get supplies*, *we make request and then it just exhausted*.*”*

– Management/Headquarters Staff

#### Post-Intervention monitoring

As post-intervention monitoring was included as part of the quantitative impact evaluation, we do not report on it here.

## Discussion

In this qualitative research, we conducted 13 semi-structured interviews with 26 key informants including management/headquarters and field team staff implementing CATI in cholera response activities in Northeast Nigeria. We compared informant responses on actual CATI implementation with planned organizational SOPs. We found that while both SI and ACF generally adhered to their SOPs during CATI implementation, deviations were made to account for contextual factors. In particular, incomplete line lists, high population density, inadequate staffing for case load, and supply stock outs greatly hindered teams’ ability to access case households and appropriately target neighbor households according to the SOPs. Many of these key challenges identified herein align with other research on NGO-led cholera outbreak responses, including need for adequate staffing, supplies, staff training, mechanisms to enable reaching case households, and coordination [12,13,14].

These qualitative findings prompt the following recommendations. First, organizations should strongly consider employing context-specific radii taking into account population density, human resources, and supplies. The largest deviation from SOPs in this study occurred in neighbor household selection, with informants highlighting the impracticality of a 150-meter radius especially in population-dense communities. Successfully employing a 150-meter radius in an urban environment requires a high level of staffing and supplies. Prior studies indicate that cholera interventions are most effective when implemented 30–100 meters from the case household, with the greatest risk of cholera transmission occurring within 50 meters [6,15]. While implementing a 150-meter radius may reach additional households at increased risk, a smaller radius may offer a more balanced option in resource-constrained contexts like humanitarian settings. Teams should thus consider reducing their chosen CATI radius, particularly in areas of high population density.

Second, while discrepancies between SOPs and implementation will often arise due to unforeseen events and challenges, more explicit guidance in SOPs on how to approach predictable context-specific challenges would better support coordinated and measurable interventions. KIIs revealed that when faced with different contexts (e.g. high population density), teams sometimes adopted incongruous approaches. Such incongruity can hinder both the effectiveness of interventions as well as the ability to adequately measure the impact of certain strategies. Additionally, informal adjustments to SOPs mid-response sometimes contributed to confusion among teams. Organizations should therefore evaluate predictable challenges (including security, population density, sociocultural norms, and supply and staff availability) in the preparedness stage and provide explicit instructions in context-specific CATI SOPs on how to best deviate in those circumstances.

Within feasible grounds, responding agencies should ensure sufficient staffing, training, and transportation to successfully implement the SOP throughout all outbreak phases. This includes having the capacity to conduct quality control to monitor and adjust implementation as needed. Multiple mechanisms should be explored during cholera emergency preparedness discussions to ensure surge capacity throughout the outbreak, especially during the peak. For example, working with national RRTs is a good first step but additional collaboration with other organizations or recruitment strategies may also be necessary to support staffing needs during high case loads. Similarly, responding agencies should ensure improved supply chain management where feasible to reduce stock outs. Prepositioning supplies and strengthening procurement mechanisms before anticipated cholera outbreaks can reduce stock out likelihood.

Furthermore, CATIs would benefit from improvements in surveillance and data management. In particular, line lists should provide multiple ways to locate a case household to reduce loss-to-follow-up (e.g. address/descriptor, phone number). Mapping technologies can also assist teams in covering all households within the radius or in avoiding overlapping rings. The challenges that informants noted in accessing line lists also underscore the need for improved coordination between CATI teams and CTCs in surveillance efforts. When a response has multiple stakeholders, each with their own SOPs, a standard process to access line list data becomes more critical. In humanitarian settings, the Health and WASH Clusters can help coordinate a cohesive CATI response [16].

Lastly, while we could not gauge the exact degree to which case households refused CATIs, reports of cases rejecting or even actively avoiding CATI teams suggest opportunities to improve program acceptance within the community. Responding agencies should adopt evidence-based risk communication and community engagement strategies to reduce the likelihood of CATI rejection.

Limitations of this work include that only 13 interviews, involving 26 individuals, were conducted. While this is not a large number of participants, it included all key CATI team personnel. These interviews were conducted remotely online due to the COVID-19 pandemic and regional insecurity, and there were occasional internet connection interruptions that challenged interview flow. The remote and paired interview structure also made it relatively difficult to encourage quieter informants to speak. Interviews were only conducted with CATI implementers. Feedback from CATI beneficiaries and partners (e.g. CTC staff) is an opportunity for future research. Additionally, due to the qualitative nature of these data, the exact source and extent of challenges such as stock outs or rejection cannot be ascertained. Lastly, the results are susceptible to bias, as the overall study may have influenced CATI implementation. Interviewees may also have been inclined to respond in ways they thought the research team wanted to hear or that positively portrayed their team. These limitations are not considered to have impacted the results presented herein.

The research presented herein represents the qualitative arm of a multi-pronged research study, and thus only provides subjective perceptions of CATI implementers. These qualitative findings provide important context in the forthcoming quantitative analysis. For example, reports that CATI teams were unable to visit all households indicate the analysis may find lower than expected ring coverage, or lower percentage of households in a ring visited by CATI teams. Similarly, some rings may have a smaller observed radius (as suggested by the challenges teams faced in reaching the full 150-meters), while other rings may see a larger radius (as suggested by the proxy measure of 300 steps which can be over 150 meters depending on a person’s stride). Furthermore, the degree to which supply shortages were mentioned during the interviews suggests the quantitative analysis could find reduced percentages of neighbor households receiving full supplies. Lastly, data on households refusing CATIs would quantify the extent to which refusals impacted CATI response. Paired with the implementation considerations learned from these interviews, the forthcoming quantitative findings may provide new insights to help guide future CATI implementation in humanitarian settings.
